# Multidisciplinary Pain Management of Chronic Back Pain: Helpful Treatments from the Patients’ Perspective

**DOI:** 10.3390/jcm9010145

**Published:** 2020-01-05

**Authors:** Timo A. Nees, Ernst Riewe, Daniela Waschke, Marcus Schiltenwolf, Eva Neubauer, Haili Wang

**Affiliations:** 1 Clinic for Orthopedics and Trauma Surgery, Center for Orthopedics, Trauma Surgery and Spinal Cord Injury, Heidelberg University Hospital, 69118 Heidelberg, Germany; ernst.riewe@med.uni-heidelberg.de (E.R.); daniela.waschke@marienhaus.de (D.W.); eva.neubauer@med.uni-heidelberg.de (E.N.); wang@zar-mannheim.de (H.W.); 2 Klinik für Gynäkologie und Geburtshilfe, Marienhaus Klinikum Hetzelstift Neustadt/Weinstraße, 67434 Neustadt an der Weinstraße, Germany; 3 Zentrum für ambulante Rehabilitation Mannheim GmbH, 68309 Mannheim, Germany

**Keywords:** multidisciplinary pain treatment, back pain, physical therapy, patient satisfaction

## Abstract

Multidisciplinary pain management programs (MPMP) for patients suffering from chronic back pain include a variety of treatment modalities. The patients’ perceived helpfulness of these treatment modalities remains unclear. The aims of this prospective observational cohort study were to assess (i) the patients’ perceived helpfulness of different treatment modalities, (ii) the influence of sociodemographic characteristics on the patient’s perspective and (iii) whether treatment outcomes are affected by helpfulness ratings. Treatment modalities of this three-week MPMP consisted of individual physiotherapy, group-based physiotherapy, relaxation therapy, aquatic therapy, back education, medical training therapy, biofeedback, psychological pain therapy and music therapy. The study comprised 395 patients. The main outcome was the patients’ perceived treatment helpfulness at the end of the program measured by a self-reported questionnaire ranging from 1 (not at all helpful) to 6 (extremely helpful). Secondary outcomes were treatment effects on pain, pain related disability, functional ability and level of depressive symptoms measured by self-reported questionnaires (NRS, PDI, FFbH-R, ADS-L). A total of 276 patients (22–64 years, 57% female) were available for overall analysis. Multivariate-analysis-of-variance- (MANOVA-) related results revealed that perceived treatment helpfulness (range 1–6) differed significantly between treatment modalities: individual physiotherapy (M = 5.00), group-based physiotherapy (M = 4.87), relaxation therapy (M = 4.6), aquatic therapy (M = 4.54), back education (M = 4.43), medical training therapy (M = 3.38), biofeedback (M = 3.31), psychological pain therapy (M = 3.15), music therapy (M = 3.02). Pain, pain related disability and levels of depressive symptoms significantly improved after the program (*p* < 0.001) whereas functional ability decreased (*p* < 0.01). Significant correlations were found between helpfulness ratings and sociodemographic data indicating that perceived treatment helpfulness was influenced by patient-related factors. Importantly, the degree of pain-related improvements was affected by the patients’ perceived treatment helpfulness. In conclusion, patients’ perceived treatment helpfulness differs significantly between treatment modalities and corresponds to treatment outcome.

## 1. Introduction

As early as 30 years ago, the Swedish orthopedic surgeon Dr. Alf Nachemson described education, exercise and encouragement as the three essential pillars of non-surgical treatment for localized back pain [1]. Indeed, numerous studies have provided profound evidence that patients suffering from chronic musculoskeletal pain benefit from physical exercise [2]. For example, exercise therapy significantly reduced pain and disability in patients with chronic low back pain when compared to usual care as revealed by systematic reviews [3,4]. Furthermore, multiple studies have reported the effectiveness of behavioral treatments for patients with chronic low back pain (see the Cochrane Back Review Group [5] for review). Therefore, treatment programs addressing both the physical and psychosocial components of pain seem to be a promising approach to reduce pain and disability. According to the biopsychosocial model of chronic pain, in multidisciplinary pain management programs (MPMP) a variety of specialists from several disciplines including physicians, psychologists, physiotherapists and occupational therapists form a multidisciplinary team to manage chronic pain problems by sharing the same philosophy, goals and treatment plans. Thus, patients can benefit from well-coordinated treatment modalities to reach the overall goals: improvements in emotional and physical functioning, pain reduction and coping [6]. Indeed, intensive multidisciplinary biopsychosocial rehabilitation reduces pain and improves function in patients with chronic back pain (CBP) when compared to non-multidisciplinary treatment or standard care [7,8,9,10]. To date, multidisciplinary biopsychosocial treatment approaches are considered the most effective means for treating chronic musculoskeletal pain [11]. Nevertheless, it is still unclear which of the various treatment modalities of MPMP are perceived as helpful by the patients. Since patient satisfaction correlates with treatment compliance and outcome [12,13,14,15], the perceived helpfulness of treatment modalities could influence treatment efficacy. Thus, we hypothesized that patients perceive different treatment modalities of a MPMP as differently helpful and that helpfulness ratings are associated with certain sociodemographic characteristics. Furthermore, we hypothesized that the perceived helpfulness of treatment modalities is related to treatment efficacy. Therefore, the primary aim of the current study was to investigate (i) which treatment modalities of a MPMP are perceived as helpful by patients suffering from CBP. Additionally, we evaluated (ii) if patient-related characteristics influence the perceived helpfulness of different treatment modalities and (iii) if pain reduction and increases in physical and psychosocial functioning is affected by the patient perceived treatment helpfulness.

## 2. Experimental Section

### 2.1. Study Design

To achieve these goals a prospective, observational cohort study was performed. The study was part of clinical care and conducted in accordance with the local ethics committee of the Medical Faculty of the University of Heidelberg and the Declaration of Helsinki and was approved by the institutional review board (257/2002).

### 2.2. Patients

A total of 395 patients with chronic non-specific localized musculoskeletal back pain completed a three-week MPMP as described below. All patients were referred to our clinic for biopsychosocial pain management due to failure of conventional treatment approaches including standard care and non-multidisciplinary interventions. Participating in the MPMP was part of clinical care. Prior to program and study enrolment, all patients were examined by an experienced physician of the pain department and eligible patients provided written informed consent. Data were obtained using paper-and-pencil self-administered questionnaires without assistance of study personnel at the beginning (T0, day 1) and at the end of the program (T1, three weeks later). From the 395 patients that had agreed to voluntarily provide self-reported data during clinical care, 331 fully completed all questionnaires at the beginning of the program. At the end of the program, 276 patients were available who had filled in the questionnaires completely at both T0 and T1.

### 2.3. Inclusion Criteria

Patients between 18 and 65 years suffering from chronic non-specific and localized back pain (>6 weeks) were included. Moreover, physical abilities allowing for daily exercises (no limitation of ordinary physical activity e.g., ability to walk 1 km) and adequate knowledge of the German language were required to participate in this study. If necessary, physical capacity was evaluated by a cardiologist in outpatient care prior to enrollment.

### 2.4. Exclusion Criteria

Patients with specific back pain including pain following fractures, cancer, systemic inflammatory diseases or infections (e.g., spondylodiscitis) as well as rheumatic diseases and severe degenerative alterations were excluded. Structural changes of the spine with acute clinical symptoms such as spinal stenosis or spondylolisthesis resulting in spinal claudication as well as disc herniation causing corresponding radicular pain also led to exclusion of this study. Furthermore, patients with multiple pain locations (e.g., chronic wide spread pain, fibromyalgia syndrome) and serious medical conditions (e.g., cardiopulmonary, vascular) were not included. Ongoing litigation or receiving worker’s compensation benefits related to their pain were also considered exclusion criteria.

### 2.5. Multidisciplinary Pain Management Program

The pain management program was an outpatient program offered at the multidisciplinary pain center of the Clinic for Orthopedics and Trauma Surgery at Heidelberg University Hospital. It consisted of three weeks of multidisciplinary treatment five days per week, five hours per day, resulting in a total of 75 treatment hours. During the multidisciplinary treatment (5 h/d) patients stayed at the pain center and were released to their private accommodation after completing their daily schedule. Treatment costs were covered by internal university funding. Integrating physical exercises, patient education and psychosocial interventions the multidisciplinary program addressed the biological, social and psychological factors of pain. By promoting physical activity and reconditioning, reducing maladaptive cognitions and improving emotional functioning as well as by building/improving coping strategies, the program aimed at restoring physical and psychosocial functioning to decrease the impact of pain on daily activities. In general, the entire multidisciplinary team used a cognitive-behavioral and psychodynamic approach to establish and increase the patients’ understanding of the nature of pain. Team meetings were held once per week to evaluate the patients’ progress and to discuss how to proceed with individual psychosocial conflicts. Treatments were delivered in groups (max. 12 patients) as well as in individual sessions (one-on-one) and are described in detail below.

#### 2.5.1. Physiotherapy

At study entry, a comprehensive physical examination was performed by the supervising physiotherapist to assess the patients’ individual physical performance including muscle strength, flexibility and endurance. Based on physical abilities individual therapeutic goals were defined in agreement with the patient and addressed in individually- and group-delivered physiotherapy sessions. An operant behavioral approach was chosen to encourage functional improvements. Group sessions included aerobic and resistance exercises. In particular, the patients participated in daily sessions of Nordic walking (1 h), core stability and stretching exercises (1 h) and in indoor/outdoor sports sessions with the opportunity for recreational activities (e.g., table tennis, badminton or soccer). Individual physiotherapy sessions (2 × 30 min/week) addressed the patients’ musculoskeletal discomfort by using patient-tailored stretching, strengthening and relaxation techniques supervised by a professional physiotherapist with experience in the treatment of CBP.

#### 2.5.2. Aquatic Therapy

Daily sessions (45 min of aquatic therapy with subsequent voluntary swimming (15 min) in a heated therapy pool were part of the program. Under the supervision of a qualified aquatic therapist, the patients performed active aquatic exercises including water aerobics and aqua running to improve physical functioning.

#### 2.5.3. Medical Training Therapy

To increase muscular strength and endurance, the patients performed device-assisted exercises (medical training therapy) in the hospital’s gym. Following a detailed initial instruction for appropriate use of the medical training devices and individual adjustment recommendations by a specialized physiotherapist, patients trained autonomously. At all times, the physiotherapist in charge was present to give assistance when necessary.

#### 2.5.4. Biofeedback Training

To improve body awareness and develop strategies of tension management, electromyogram-based biofeedback training was performed once a week for 45 min. Muscle tension was assessed at the neck and lower back with surface electrodes measuring muscle activity. Acoustic and visual feedback was provided and relaxation techniques to reduce muscle tension were applied. The relationship between muscle tension, stress and pain was explained and patients were asked to practice the instructed relaxation techniques at home. Moreover, we encouraged the patients to develop their own strategies to reduce tension.

#### 2.5.5. Back Education

Besides patient education on basic spine anatomy and function, back education (2 h/week) included exercises to increase strength and endurance of essential core stability muscles such as the transversus abdominis, multifidus and pelvic floor.

#### 2.5.6. Relaxation Therapy

Group relaxation sessions took place twice a week for one hour each. Relaxation therapy included Jacobson’s progressive muscle relaxation and autogenic training and was conducted by a qualified psychologist.

#### 2.5.7. Music Therapy

Patients participated in group music therapy once a week for one hour. Supervised by an approved music therapist, active music therapy was performed using instruments and voices.

#### 2.5.8. Psychological Pain Therapy

Psychological pain therapy consisted of group and individually-delivered interventions. Important elements of group sessions (1.5 h/week) were psychoeducational training including behavioral therapeutic techniques, problem solving strategies and stress management. One-on-one psychological sessions (1 h/week) aimed at identifying and addressing the patient’s individual psychosocial factors and emotional conflicts contributing to the development and persistence of CBP.

#### 2.5.9. Medical Supervision

To ensure adequate medical supervision, appointments with the attending physicians (physicians specialized in physical medicine and rehabilitation or orthopaedics) were scheduled twice a week for one hour. Besides physical examinations at study entry and at end of the program the physicians monitored drug usage and managed potential medical issues occurring during the three weeks of treatment. In general, patients were encouraged to reduce/withdraw their pain medication under medical supervision. None of the patients needed intravenous or intramuscular analgesics either at study entry or during the program. When necessary, non-opioid analgesics, including nonsteroidal anti-inflammatory drugs, Metamizole or Acetaminophen, were prescribed. Patients using oral Opioids at study entry were encouraged to reduce the opioid intake under medical supervision. Additional opioids were not prescribed.

After completing the program, patients were discharged without further interventions by the pain department. They were allowed to contact the referring physician but were advised to independently cope with future pain episodes applying the acquired knowledge and techniques without seeking immediate medical attention. Health care utilization following the end of the program was not monitored.

### 2.6. Measurements/Outcome Parameters

#### 2.6.1. Sociodemographic and Baseline Clinical Data

Sociodemographic data were recorded prior to the program (T0) using the self-reported standardized questionnaire of the German Pain Society [16] and included gender, age, marital status and educational information as well as baseline clinical data such as body mass index, smoking status and regular sports activity. Additionally, the stage of chronic pain was assessed using the Mainz Pain Staging System (MPSS) [17]. Besides temporal aspects of pain, the MPSS includes pain locations (lower back pain/upper back pain/lower + upper back pain), frequency of analgesic intake (no/sometimes/regularly/daily) and the patients’ utilization of the healthcare system to evaluate the grade of pain chronicity from I-III. The MPSS was validated for several pain syndromes including chronic low back pain [18,19]. Its clinical relevance, predictive validity and criterion validity with regard to changes in pain, function or depression-related parameters (NRS, FFbH-R and ADS-L) are outlined by Hampel & Moergel in 2008 [18].

#### 2.6.2. Primary Outcome Measure: Patients’ Perceived Treatment Helpfulness

To explore which treatments of the MPMP (the program in general, individual physiotherapy, group-based physiotherapy, aquatic therapy, medical training therapy, back education, biofeedback, relaxation therapy, music therapy, psychological pain therapy) are perceived as helpful patients assessed the different treatments regarding pain improvement using a self-reported questionnaire at the end (T1) of the program. They evaluated how helpful respective treatments have been in reducing their pain on a scale from 1 to 6 (1 = not at all helpful, 2 = I don’t know, 3 = slightly helpful, 4 = moderately helpful, 5 = very helpful, 6 = extremely helpful). Treatments were considered helpful when rated ≥ 4 (= moderately helpful).

#### 2.6.3. Secondary Outcome Measures

To detect treatment effects after three weeks, analgesic intake, pain intensities (numeric rating scale = NRS), pain-induced disability (Pain Disability Index = PDI), functional capacity (Hannover Functional Ability Questionnaire = FFbH-R) as well as depression and depressive disorder (German Version of the Center for Epidemiologic Studies Depression Scale = ADS-L) were monitored throughout the study at both T0 and T1.

##### Pain Intensity (NRS)

Pain intensity was rated on an 11-point numeric rating scale (NRS 0–10) with terms describing pain severity extremes (0 = “no pain at all”; 10 = “worst pain imaginable”). Patients rated their current as well as average, worst and least pain within the last week. In current literature, the self-reported NRS is considered a reliable measure to assess pain intensities whereas a 1.5 to 2-point minimum detectable change (MDC) seems to be necessary for clinical meaningfulness [20,21,22,23]. Consistently, improvements < or = 1.5 points are deemed irrelevant [20,21]. Importantly, the level of changes in self-reported pain seems to depend on initial pain ratings assessed at baseline. In contrast to higher levels, lower baseline levels of pain result in lower improvements at follow-up [21,22].

##### Pain Disability Index (PDI)

The German version of the Pain Disability Index (PDI) was used to measure pain-related disability [24,25,26]. This valid and reliable self-report instrument evaluates the impact of chronic pain on seven activities of daily living (items). The degree to which pain interferes with daily activities is rated on a scale from 0 (min. disability) to 10 (max. disability). Two factors are measured: 1 = voluntary activities (PDI items 1–5); 2 = obligatory activities such as self-care and life support activity (PDI items 6–7). The aggregate value (0–70) is multiplied by 10 and divided by the number of completed items (max. 1 missing item) to express the percentage of overall disability ranging from 0 (minimal pain-related disability) to maximal disability levels of 100%. PDI scores > 30% are considered as clinically relevant. The internal consistency (Cronbach’s α) of the PDI has been calculated previously. For factor 1, Cronbach’s *α* was 0.85, and for factor 2, Cronbach’s *α* was 0.70 [26]. The one-week test-retest reliability was considered good (ICC = 0.91) and intercorrelation analyses to VAS (Visual Analogue Scale)/NRS showed r values of 0.41 [27] The minimal clinically important change (MCIC) was also assessed for both factors of the PDI (factor 1: MCIC = 9.5 points, sensitivity 0.74, specificity 0.70; factor 2: MCIC = 8.5 points, sensitivity 0.74, specificity 0.70) [28]. Therefore, depending on the factor that was used as the external criterion, a change of 8.5 to 9.5 points may be considered a MCIC. Total responsiveness regarding receiver operating characteristic (ROC) curve analyses was considered good (area under the curve (AUC) < 0.70) although the subscale of obligatory activities was not responsive [28].

##### Functional Ability (FFbH-R)

Back function was assessed using the Hannover Functional Ability Questionnaire for measuring back-pain-related functional status (FFbH-R) [29]. The FFbH-R is a self-reported measurement tool to evaluate the functional impact of pain on activities of everyday life. In this 12-item questionnaire (max. two missing items) the patients assess their functional capacity to perform activities of daily living resulting in an aggregate value between 0 (min. functional capacity) and 100 (max. functional capacity). Scores from 100 to 80% and from 79 to 70% indicate normal functional ability and moderately reduced capacity, respectively. In contrast, values between 69 and 60% are considered abnormal and FFbH-R scores < 59% indicate clinically relevant decreases in functional ability. The internal consistency of the FFbH-R is considered very good (Cronbach α = 0.90). The item-intercorrelation and the one-week test-retest reliability were found to be r = 0.5 and ICC < 0.75, respectively [29]. The discriminative ability of the FFbH-R as well as the dichotomized measures of MCIC via ROC-curve analyses were outlined in a multidisciplinary setting of a Norwegian prospective cohort study (MCIC = 0.8, AUC = 0.74, sensitivity = 0.69, specificity = 0.78, 95% confidence interval = 0.58 to 0.90) [30].

##### German Version of the Center for Epidemiologic Studies Depression Scale (ADS-L)

To evaluate the current level of depressive symptoms, the German equivalent of the Center for Epidemiologic Studies Depression Scale (ADS-L) [31] was applied before and after the MPMP. The ADS-L- consists of 20 items (max. two missing items) with a total score ranging from 0 to 60. Increasing scores indicate higher levels of depressive symptoms and scores ≥ 23 are considered clinically relevant. The internal consistency ranges from 0.82 to 0.92 (Cronbach *α*), and the one-week, two-week and three-month test-retest reliability were good (*r* = 0.63, *r* = 0.48 and *r* = 0.58, respectively). Factorial, discriminant and convergent validity has been evaluated in a variety of studies (sensitivity = 0.90; specificity = 0.87; AUC = 0.94, MDC = 5 points) [31].

### 2.7. Statistical Analysis

All analyses were performed using IBM SPSS^®^ Statistics 24.0 (Armonk, NY, USA) for Microsoft^®^ Windows V.10 (Redmond, WA, USA) with a significance level of 5% (α = 0.05) and a confidence interval (CI) set to 95%. Only data from patients completing the questionnaires at both T0 and T1 were used. As a first step, an explorative data analysis for all variables was carried out. Normal distributions for subsequent correlation analyses and *t*-tests were assessed by the Kolmogorov–Smirnov and the Shapiro–Wilk test. The homogeneity of variance according to MANOVA-related calculations was verified by the Levenne test.

The average perceived helpfulness of different treatments was calculated and expressed as mean ± SD. Furthermore, Pearson’s r or its non-parametric counterpart Spearman’s rho were applied to assess whether perceived treatment helpfulness is influenced by sociodemographic characteristics at study entry. In addition, all helpfulness variables were dichotomized (not helpful/helpful) using a cut-off ≥ 4 (moderately helpful). To assess if potential relationships exist between the patients’ perceived helpfulness and analgesic intake, pain intensities (NRS), functional ability (FFbH-R), pain-related disability (PDI) and manifestation of depressive symptoms (ADS-L) the following three steps were carried out. At first, either paired *t*-tests or non-parametric Wilcoxon signed rank tests were used to detect significant changes in NRS, FFbH-R, PDI and ADS-L after completing the program. For variables that significantly decreased or increased, differences between T0 and T1 were calculated as delta values (Δ) in the second step. Except for Δ FFbH-R (as change in percent), a positive assignment for improvements was achieved by subtracting T1 from T0 values (Δ analgesics intake, Δ pain intensities as change in NRS, Δ PDI as change in percent, Δ ADS-L as change in the sum score). Finally, using the delta values as dependent variables, one-way MANOVA with Bonferroni adjustments was conducted. If the homogeneity of variance was violated, Kruskal–Wallis tests were used instead. CIs and effect sizes (Partial-Eta-squared = η^2^) are provided by MANOVA-related calculations. As presented in Table 1, data interpretations are based on recommendations by Jacob Cohen [32]. For non-parametric calculations (Wilcoxon or Kruskal–Wallis tests) η^2^ was calculated using an online tool [33] based on work from Cohen [34] and Fritz, Morris and Richler [35].

## 3. Results

### 3.1. Sociodemographic and Pain-Related Characteristics at Baseline

A total of 276 patients completed the questionnaires at both T0 and T1. The sociodemographic characteristics of this study population are presented in Table 2.

Prior to treatment, mean pain intensity within the last week was rated 5.2 (5.24 ± 2.08), whereas worst and least pain was evaluated 7 (6.97 ± 2,13) and 2.8 (2.77 ± 1.93) on the NRS, respectively. On average, current pain at T0 was rated 4.4 (4.41 ± 2.42). Occasional use of analgesics was stated by 32% of the patients, whereas 14% and 23% reported analgesic intake on a regular basis and daily, respectively. Mean functional capacity (FFbH-R) at T0 was 75% (74.54 ± 16.89) which represents moderately reduced functional capacity (range 79–70). Normal functional ability was presented by 41% of the patients (range 100–80%) and nearly a quarter of the study population (23.6%) showed clinically relevant decreases in functional ability (range 59–0%). Mean pain-related disability (PDI) was 27% (26.7 ± 12.27) and clinically relevant disabilities were observed for 36% of this study population (range 30 to 100% = clinically relevant). For the ADS-L, clinically relevant depressive symptoms (ADS-L score ≥ 18) were present in 47% of the participants with 19% suffering from depression (ADS-L score > 27). The mean ADS-L score was 19.43 ± 9.53. Detailed information about pain-related characteristics are outlined in Table 3.

### 3.2. The Patients’ Perceived Treatment Helpfulness at Discharge

**Figure 1:**
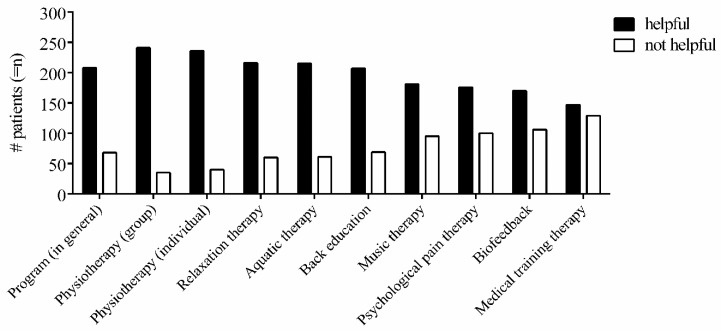
The patients’ perceived treatment helpfulness at discharge. The number (#) of patients (= *n*) rating respective treatments of the multidisciplinary pain management program as being helpful (blue) or not helpful (red). Treatments were considered as helpful when rated ≥ 4 (= moderately helpful) and as not helpful when rated < 4 (rating scale from 1–6).

As presented in Figure 1, 75% (*n* = 208) of the patients completing the program rated the MPMP in general as helpful. Mean perceived helpfulness was 4.39 ± 1.23.

Among the different treatments, the vast majority of patients considered group-based physiotherapy (PTg, *n* = 241, 87.3%) and individual physiotherapy as being helpful (PTi, *n* = 236, 85.5%). On average, both group- and individual-based physiotherapy were perceived as very helpful (PTg: 4.87 ± 1.14, PTi: 5.0 ± 1.2). Importantly, almost half of the patients (*n* = 134, 48.6%) reported that individually-delivered physiotherapy was extremely helpful to alleviate CBP. Similarly, a high percentage of patients (*n* = 106, 38.4%) also rated group-based physiotherapy as extremely helpful. Following physiotherapy, most of the patients considered relaxation therapy (*n* = 216, 78.3%), aquatic therapy (*n* = 215, 77.9%) and back education (*n* = 207, 75.0%) as beneficial. The mean perceived helpfulness of relaxation therapy (4.6 ± 1.27), aquatic therapy (4.54 ± 1.37) and back education (4.43 ± 1.3) was moderately or very helpful. In contrast, the mean helpfulness of medical training therapy (3.38 ± 1.51), biofeedback (3.31 ± 1.48), psychological pain therapy (3.15 ± 1.42) and music therapy (3.02 ± 1.47) ranged between slightly and moderately helpful. Less than two-thirds of the patients consider medical training therapy (*n* = 147, 53.3%), biofeedback (*n* = 170, 61.6%), psychological pain therapy (*n* = 176, 63.8%) or music therapy (*n* = 181, 65.6%) to be beneficial in reducing CBP.

**Figure 2:**
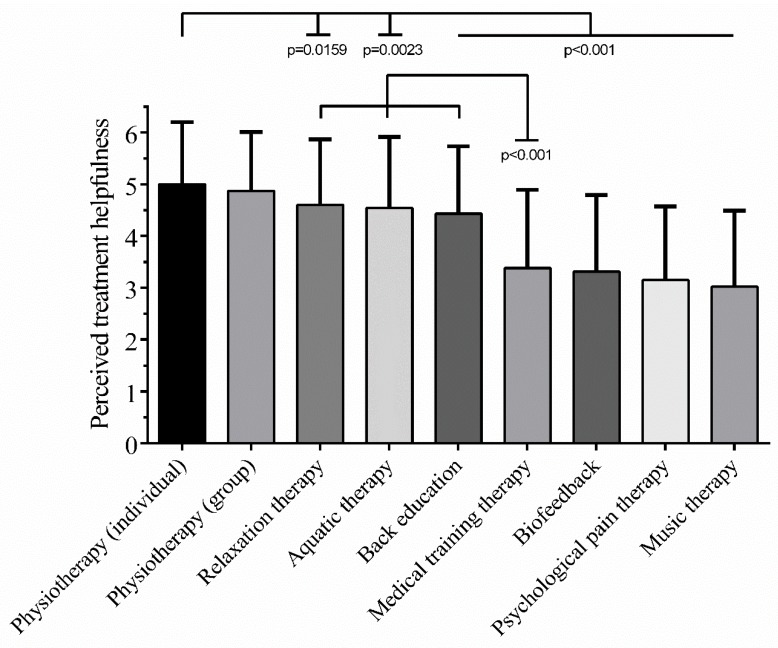
Mean perceived treatment helpfulness. Mean perceived helpfulness differs significantly between treatment modalities of the multidisciplinary pain management program (Analysis of variance (ANOVA): *p* < 0.001). Mean helpfulness ratings (±SD) ranged from 5 (±1.2) for individually-delivered physiotherapy to 3.02 (±1.47) for music therapy. Highly significant differences were observed between individually-delivered physiotherapy and back education, medical training therapy, biofeedback, psychological pain therapy as well as music therapy (Tukey’s multiple comparison: *p* < 0.001). Moreover, relaxation therapy, aquatic therapy and back education were perceived to be significantly more helpful than medical training therapy (Tukey’s multiple comparison: *p* < 0.001). Table 1 of the program (1 = not at all helpful, 2 = I don’t know, 3 = slightly helpful, 4 = moderately helpful, 5 = very helpful, 6 = extremely helpful). Data is presented as mean ± standard deviation.

In summary, mean patient-perceived helpfulness ranged from 5 (±1.2) for individually-delivered physiotherapy to 3.02 (±1.47) for music therapy (Figure 2). In fact, mean helpfulness of the different treatments was ranked as follows: (1) individual physiotherapy; (2) group-based physiotherapy; (3) relaxation therapy; (4) aquatic therapy; (5) back education; (6) medical training therapy; (7) biofeedback; (8) psychological pain therapy; (9) music therapy. Detailed information (frequencies, means, percent, SEM, SD) are provided in Tables S1 and S2.

### 3.3. Influence of Sociodemographic Characteristics on the Patients’ Perceived Helpfulness

Pain location did not influence the perceived helpfulness of different treatments. Neither patients with lower or upper back pain nor participants suffering from both showed differences in their perspective on treatments helping to alleviate CBP. Similarly, smoking status was not correlated with the patients’ perceived helpfulness.

Significant but generally weak correlations with small effect sizes (Spearman’s rho) were found between several treatments and sociodemographic/pain-related characteristics (Table S3). A reciprocal correlation exists between pain chronicity and perceived helpfulness of the MPMP in general (r_s_ = −0.158; *p* = 0.009) indicating higher helpfulness ratings for patients with a lower level of chronicity. Furthermore, a weak correlation was found between chronicity status and back education treatment (r_s_ = −0.120; *p* = 0.046) suggesting that patients with higher stages of chronic pain might favor back education training.

A significant correlation was also observed between age and psychological pain therapy (r_s_ = 0.147; *p* = 0.015) indicating that older patients tend to consider psychological interventions as more helpful. In contrast, the older the patient the less helpful device-assisted strength training (medical training therapy) was perceived (r_s_ = −0.189; *p* = 0.002).

Moreover, there were weak but significant correlations between the level of education and the perceived helpfulness of medical training therapy (r_s_ = 0.133, *p* = 0.028), psychological pain therapy (r_s_ = −0.153, *p* = 0.011) and music therapy (r_s_ = −0.132, *p* = 0.029). This suggests that the higher the education, the more likely patients were to perceive medical training therapy as helpful. The lower the education, the more likely patients were to perceive psychological and music therapies as helpful.

Patients with higher BMI scores rated aquatic (r_s_ = 0.148, *p* = 0.014) and psychological pain therapies (rs = 0.122, *p* = 0.043) as more helpful than did those with lower BMI scores.

In addition, the lack of regular sporting activity prior to the program correlates with increasing helpfulness ratings for medical training therapy. Thus, patients who did not participate in regular sports activities before study entry seem to perceive medical training therapy as being helpful to reduce CBP (r_s_ = −0.139, *p* = 0.021) when compared to patients who worked out on a regular basis.

### 3.4. The Multidisciplinary Pain Management Program Reduces Pain-Related Complaints

Table 4 highlights the changes of pain-related outcome parameters during the three-week MPMP. As illustrated, pain intensities (NRS), pain related disability (PDI) and levels of depressive symptoms (ADS-L) were significantly reduced after completing the program (T1) when compared to study entry (T0). For detailed information about the results of the Wilcoxon signed-rank test including Z-values, see Table S4.

Except for the FFbH-R, large effect sizes for changes in pain-related outcome parameters were observed. Mean pain intensity decreased from 5.24 ± 2.08 at study entry to 3.80 ± 2.02 (Z = −10.164, *p* < 0.001, η^2^ = 0.374). Similarly, worst, least and current pain significantly improved from 6.97 ± 2.13 to 5.8 ± 2.35 (worst pain Z = −8.204, *p* < 0.001, η^2^ = 0.244), 2.77 ± 1.93 to 1.95 ± 1.85 (least pain Z = −8.042, *p* < 0.001, η^2^ = 0.234) and from 4.41 ± 2.42 to 3.07 ± 2.37 (current pain Z = −9.134, *p* < 0.001, η^2^ = 0.302), respectively. Besides pain intensities, a 10% decrease from baseline was observed for the PDI score (T0: 26.7 ± 12.27; T1: 17.4 ± 11.78; Z = −12.387, *p* < 0.001, η^2^ = 0.556) and the mean ADS-L score dropped significantly from 19.43 ± 9.53 to 9.77 ± 8.27 (Z = −12.592, *p* < 0.001, η^2^ = 0.574). Treatment-induced changes in average pain intensity, pain induced disability (PDI) and depressive symptoms (ADS-L) were clinically meaningful, demonstrating the efficacy of the program. Interestingly, despite these improvements in pain-related complaints functional ability (FFbH-R) significantly decreased with a medium sized effect from 74.54% ± 16.89 to 71.49% ± 15.07 (Z = −4.198, *p* < 0.01, η^2^ = 0.064) during the study. Analgesic intake was not influenced by the program.

### 3.5. The Patients’ Perspective Corresponds to Pain-Related Treatment Outcomes

To assess associations between the subjective evaluation of treatment helpfulness by the patients and changes in objectively measured outcome parameters, helpfulness ratings were dichotomized (≥ 4 helpful vs. < 4 not helpful) and analyzed in relation to changes in pain-related treatment outcome. Accordingly, Table 5 illustrates the effects of the dichotomized patients’ perceived treatment helpfulness on changes (delta Δ) in pain, physical and psychosocial functioning. For more detailed information regarding the statistical analyses, see Tables S5–S8.

Patients reporting that the MPMP in general helped to reduce back pain indeed showed significant decreases in Δ pain values during this study compared to those considering the program as not being helpful (Δ pain average *p* < 0.001, CI = −1.431 to −0.386; Δ pain worst *p* < 0.001, CI = −1.808 to −0.674; Δ pain least *p* = 0.003, CI = −0.995 to −0.095; Δ pain current *p*= 0.004, CI = −1.461 to −0.262). Thus, the patients’ perspective on the helpfulness of different treatments significantly corresponds to the degree of pain improvement. Furthermore, perceiving the program as helpful was associated with reduced levels of pain-related disability (Δ PDI, *p* = 0.005, CI = −6.518 to −1.218). Calculated effect sizes ranged from small (η^2^ < 0.06) to medium (Δ pain average η^2^ = 0.060 and Δ pain worstη^2^ = 0.067).

Surprisingly, the analyses revealed no relationship between the perceived helpfulness of individual physiotherapy and changes in pain-related characteristics (Δ pain intensities, Δ PDI, Δ FFbH-R, Δ ADS-L). Thus, participants experiencing individual physiotherapy as helpful did not show a greater degree of improvements in pain-related parameters when compared to patients that did not consider individual physiotherapy as being helpful to reduce CBP. Strikingly, on average, individual physiotherapy was rated as very helpful and ranked highest among the treatments.

In contrast to individually-delivered physiotherapy, patients considering group-based physiotherapy as helpful in decreasing CBP showed significantly higher degrees of improvement in pain intensities and disability than patients rating group-based physiotherapy as not helpful (Δ pain average *p* < 0.001, CI = −2.084 to −0.744; Δ pain worst *p* < 0.001, CI = −2.146 to −0.666; Δ pain least *p* = 0.01, CI = −1.295 to −0.131; Δ pain current *p* = 0.006, CI = −1.854 to −0.300; Δ PDI *p* < 0.001, CI = −10.111 to −3.331). All measured effect sizes were small except of Δ pain average with a medium effect size of η^2^ = 0.069.

After completion of the program a significantly greater degree of reduction in pain intensities with small effect sizes was observed for patients rating relaxation therapy as helpful in contrast to patients that consider relaxation therapy not helpful (Δ pain average *p* = 0.015, CI= −1.235 to −0.132; Δ pain worst *p* = 0.01, CI = −1.397 to −0.188; Δ pain least *p* = 0.005, CI = −1.136 to −0.200; Δ pain current *p* = 0.005, CI = −1.530 to −0.278).

Patients reporting aquatic therapy to be beneficial in reducing pain showed a higher degree of improvement in pain worst only (*p* = 0.045, CI = −1.077 to 0.135), when compared to the 22.1% of the study population that rated aquatic therapy as not helpful. The effect size for this association was small.

The degree of improvements in pain intensities was significantly greater in patients perceiving back education as helpful treatment when compared to the 25% of the study population that rated back education as not helpful (Δ pain average *p* < 0.001, CI = −1.428 to −0.388; Δ pain worst *p* < 0.001, CI = −1.627 to −0.489; Δ pain least *p* = 0.019, CI = −0,984 to −0.089; Δ pain current *p* = 0.030, CI = −1.267 to −0.067). MANOVA revealed only small effect sizes.

For patients evaluating medical training therapy as helpful the degree of average and worst pain reduction as well as the decrease of PDI was significantly higher than for the 46.7% of the study population experiencing medical training therapy as not helpful (Δ pain average *p* = 0.016, CI = −0.979 to −0.066; Δ pain worst *p* < 0.001, CI = −1.284 to −0.289; Δ PDI *p* = 0.014, CI = −5.560 to −0.980). Again, effect sizes were small.

Significant associations with small effect sizes were found between the perceived helpfulness of biofeedback and changes in pain-related outcome parameters. Rating biofeedback as helpful was related to a higher degree of improvements in pain intensities and the PDI when compared to patients reporting that biofeedback did not help to reduce CBP (Δ average pain *p* = 0.003, CI = −1.169 to −0.238; Δ worst pain *p* = 0.003, CI = −1.276 to −0.254; Δ least pain *p* = 0.025, CI = −0.856 to −0.058; Δ current pain *p* = 0.033, CI = −1.115 to −0.046; Δ PDI *p* = 0.011, CI = −5.426 to −0.716).

With small effect sizes, improvements of worst pain intensity were significantly greater in patients considering psychological pain therapy as being helpful to ameliorate CBP when compared to participants perceiving this treatment modality as not helpful (*p* = 0.045, CI = −1.054 to −0.011).

Patients reporting that music therapy helps to alleviate CBP did not show any changes in pain-related characteristics when compared to the participants rating music therapy as not helpful.

Importantly, patients’ perspective on the helpfulness of different treatment modalities also corresponds to changes in the FFbH-R with small effect sizes. However, MANOVA-pairwise comparisons revealed that rating treatments as helpful was significantly associated with a higher degree of impairment in pain-related functional ability. Thus, considering the following treatments as not helpful corresponds to a significantly lesser decrease in back function (program in general *p* < 0.001, CI = 6.408 to 16.919; group-based physiotherapy *p* < 0.001, CI = 9.661 to 23.185; aquatic therapy *p* = 0.036, CI = 2.011 to 13.155; back education p = 0.002, CI = 3.164 to 13.792; music therapy *p* = 0.005, CI = 2.104 to 11.823; biofeedback *p* = 0.004, CI = 2.182 to 11.671; medical training therapy *p* = 0.002, CI = 3.036 to 12.246).

## 4. Discussion

To our best knowledge this is the first study assessing patients’ perceived helpfulness of different treatment modalities of a multidisciplinary pain management program in relation to sociodemographic characteristics and treatment outcome. Most importantly, the present study demonstrates that the patient perceived treatment helpfulness significantly varies between different treatment modalities. The majority of patients (75%) considered the program as being helpful. However, focusing on specific treatment components revealed substantial differences in the perceived helpfulness. From the patients’ perspective, physiotherapy—both individually- and group-delivered—was reported to be very helpful. In contrast, psychological pain therapy and music therapy were only perceived as slightly helpful. At first this might suggest that physical treatments are perceived more effective by patients than psychological treatment approaches. However, CBP patients perceive relaxation therapy and back education (more psychological interventions than physical treatments) more helpful than medical training therapy. Moreover, no difference in the perceived helpfulness was observed between group-delivered physiotherapy and relaxation therapy. Previously, it was reported that patient satisfaction assessed by the treatment helpfulness questionnaire [15] might be even greater for psychological and educational treatments than for physical therapy and medical treatment modalities as revealed by a study in three interdisciplinary pain management centers [36]. Thus, general and final conclusions regarding the helpfulness of physical and psychological treatments cannot be drawn. The helpfulness ratings of treatment modalities differ significantly between centers/programs and the comparability of these ratings between programs is limited due to differences in treatment applications and doctor/therapist-patient relationships [36]. Nonetheless, independent of the program, it is consistently evident that patients perceive several treatment modalities to be of differing helpfulness, which is in line with our findings.

To assess whether these differences in perceived helpfulness are associated with certain patient-related characteristics, correlation analyses were performed. Several significant correlations between sociodemographic characteristics and helpfulness ratings of different treatments were identified. However, correlations were consistently weak (r values < |0.2|). This indicates that individual responses to different treatment modalities are difficult to predict using sociodemographic characteristics of this study population. Similar results were reported previously [36]. Yet, sociodemographic parameters can influence pain perception and treatment effects. For example, women have a higher prevalence of chronic pain and present with more severe and frequent pain than men [37,38,39]. Furthermore, sex differences might impact the outcome of both pharmacological and non-pharmacological pain treatments [40,41]. Whereas women might benefit more from psychological interventions such as cognitive behavioral therapy [42], men might respond better to conventional physiotherapy [43]. In a multimodal pain management program including 496 patients, women showed greater improvements in pain-related characteristics than men [38]. Nevertheless, it remains elusive whether or which sociodemographic parameters, including gender, determine the differences in the perceived helpfulness of treatment modalities. Analyzing patient profiles including several patient-related characteristics rather than correlating single sociodemographic parameters might reveal groups of patients perceiving specific treatments as helpful. MPMPs to the patients’ perceived treatment helpfulness might increase patient satisfaction and treatment efficacy. According to our results the perceived helpfulness of treatment modalities corresponds to pain-related outcomes. MANOVA analysis revealed for virtually all treatment modalities significant associations between the patients’ perspective on helpful treatments and the degree of improvements in pain-related characteristics. For example, patients perceiving group-delivered physiotherapy as helpful indeed presented significantly higher improvements in pain-related outcome parameters. Even for treatment modalities that on average were only perceived as slightly helpful (medical training therapy, biofeedback and psychological pain therapy) significant relations between helpfulness ratings and improvements in pain characteristics were found. Thus, our results suggest that perceiving a certain treatment as helpful is associated with greater treatment-induced improvements in pain-related outcomes. These results, however, should be interpreted with caution. Effect sizes for the associations between helpfulness ratings and treatment effects (improvements in pain-related characteristics) are virtually all small. Furthermore, helpfulness ratings do not necessarily correspond to improvements in pain-related characteristics. There were no significant associations between the patients’ perceived helpfulness of individually-delivered physiotherapy and music therapy and improvements in pain-related outcome. Interestingly, the perceived helpfulness of individually-delivered—on average ranked the most helpful treatment of the program—was not related to ameliorations of pain-related outcome measures. Addressing the patients’ musculoskeletal discomfort with individually-tailored stretching, strengthening and relaxation techniques, patients receive particular attention from a professional physiotherapist in one-on-one sessions. Due to the individual treatment approach focusing on specific physical problems and needs the patients might experience individual physiotherapy as highly effective. Moreover, physical treatments often influence the patients’ pain experience immediately. Although treatment effects might not persist, the participants receive a physical response that can be directly linked to the therapy. In contrast, the effects of individually-delivered psychological pain therapy might not lead to immediate feedback.

Interestingly, back function decreased during the study, although all pain-related outcome measures improved with large effect sizes. Treatment-induced changes in average pain intensity, pain induced disability (PDI) and depressive symptoms (ADS-L) were clinically meaningful, demonstrating the efficacy of the program. In line with the treatment-induced improvements in pain characteristics, the MPMP in general was considered helpful in alleviating back pain. It remains unclear why the self-assessed functional capacity decreased. Mean functional ability was already moderately reduced at study entry. Potentially, due to the intensive program the patients reached their physical limits resulting in temporarily lower back function ratings. In fact, most adverse events of physical exercise in patients suffering from chronic pain are increased muscle pain and soreness which subside after a few weeks of the intervention as revealed by a systematic review [44]. Moreover, significant increases in functional ability were evident six months after a three-week MPMP, whereas no relevant changes in FFbH-R scores were observed immediately thereafter [45].

### Limitations

Long-term results of the relation between treatment helpfulness ratings and pain-related characteristics beyond the study are not available since the patients’ perceived helpfulness was not assessed in follow-up visits. Thus, changes in the patients’ perspective on helpful treatments after completing the program need to be addressed in future studies. Analyzing the association between long-term treatment effects and perceived helpfulness could reveal clinically relevant treatment modalities impacting the patients’ pain behavior.

Due to the amount of sociodemographic and pain-related parameters as well as the helpfulness ratings assessed in this study (a total of 60 variables), approximately 70% of the study population completed all questions at both T0 and T1 and a decent number of participants (*n* = 276) was available for overall analyses. Nevertheless, breaking down the integrative effect of multimodal treatments by assessing the perceived helpfulness of single treatment modalities and associations with patient-related characteristics might underestimate the overall efficacy of the MPMP. Furthermore, our results don’t include implicit treatment effects. Although perceived as activation of the body, physical treatment approaches implicitly impact pain behavior on a psychological level. Vice versa, explicitly perceived as not helpful, the implicit effect of psychological treatments is unknown and remains to be determined.

## 5. Conclusions

In conclusion, the patients’ perceived treatment helpfulness differs significantly between treatment modalities of multidisciplinary pain management programs and corresponds to the outcome of pain-related characteristics. However, factors determining the patients’ perspective on the helpfulness of different treatments remain unclear in this study population.

## Figures and Tables

**Table 1:** Interpretation of effect-sizes based on Cohen [32].

(Partial-) Eta-Squared (η^2^)	Correlation Coefficient (r_(s)_)	Interpretation
η^2^ > 0.01	r_(s)_ > = 0.10	small effect
η^2^ > 0.06	r_(s)_ >= 0.30	medium effect
η^2^ > 0.14	r_(s)_ > = 0.50	large effect

**Table 2:** Sociodemographic characteristics of the study population (*n* = 276).

Variable	Response	*n* =	Mean/Percent	SD/Range
Age		276	44.5	9.073/22–62
Gender	Female	157	56.9%	
	Male	119	43.1%	
Marital Status	Unmarried	55	19.9%	
	Married	190	68.8%	
	Widowed	3	1.1%	
	Divorced	28	10.1%	
Education	Low	104	37.7%	
	Intermediate	87	31.5%	
	High	85	30.8%	
BMI	Underweight	5	1.8%	
	Normal	134	48.6%	
	Overweight	103	37.3%	
	Obese	34	12.3%	
Smoking	Yes	176	63.8%	
	No	100	36.2%	
Sports Activity	Yes	183	66.3%	
	No	93	33.7%	
Pain Location	Lower back	157	56.9%	
	Upper back	76	27.5%	
	Both	43	15.6%	
Pain Chronicity	Grade I	86	31.2%	
	Grade II	120	43.5%	
	Grade III	70	25.4%	

SD = Standard Deviation; BMI = Body Mass Index.

**Table 3:** Pain-related characteristics of the study population (*n* = 276) at baseline (T0) and at the end of the multidisciplinary pain management program (T1).

Variable	Response	T0				T1			
		*n* =	Percent	Mean	SD	*n* =	Percent	Mean	SD
average pain last week	0 to 10	276	5.24	5.24	2.08		3.80	2.27	1.23
worst pain	0 to 10	276	6.97	6.97	2.13		5.80	3.80	2.02
least pain	0 to 10	276	2.77	2.77	1.93		1.95	5.80	2.35
current pain	0 to 10	276	4.41	4.41	2.42		3.07	1.95	1.85
analgesic intake	no	88	31.9			107	38.8		
	yes, occasionally	87	31.5			62	22.5		
	yes, regularly	39	14.1			32	11.6		
	yes, always	62	22.5			75	27.2		
FFbH-R	overall score			74.54	16.98			71.49	15.07
	normal	114	41.3			75	27.2		
	moderate	53	19.2			64	23.2		
	abnormal	44	15.9			63	22.8		
	clinically relevant	65	23.6			74	26.8		
PDI	overall score			26.70	12.27			17.40	11.79
	low disability	177	64.1			230	83.3		
	high disability	99	35.9			46	16.7		
ADS-L	overall score			19.43	9.532			9.77	8.266
	normal	146	52.9			232	84.1		
	somatoform disorder - anxious	50	18.1			19	6.9		
	depressive symptoms	27	9.8			12	4.3		
	depression	53	19.2			13	4.7		

Values rounded; *n* = number of patients; SD = Standard Deviation; FFbH-R = Hannover Functional Ability Questionnaire; PDI = Pain Disability Index; ADS-L = German Version of the Center for Epidemiologic Studies Depression Scale.

**Table 4:** The multidisciplinary pain management program reduces pain-related complaints.

Variable	T0 (*n* = 276)			T1 (*n* = 276)				
	Mean	SD	Median *	Mean	SD	Median *	*p*-Value	η^2^
Pain average last week	5.24	2.079	2 (IQR 1.00; 3.00)	3.80	2.021	4 (IQR 2.00; 5.00)	<0.001	0.374
Pain worst	6.97	2.127	8 (IQR 6.00; 9.00)	5.80	2.350	6 (IQR 4.00; 8.00)	<0.001	0.244
Pain least	2.77	1.930	3 (IQR 1.00; 4.00)	1.95	1.846	1 (IQR 1.00; 3.00)	<0.001	0.234
Pain current	4.41	2.416	4 (IQR 3.00; 6.00)	3.07	2.374	3 (IQR 1.00; 5.00)	<0.001	0.302
FFbH-R	74.54	16.887	75 (IQR 63.00; 88.00)	71.49	15.070	71 (IQR 58.00; 83.00)	<0.001	0.064
PDI	26.70	12.266	25 (IQR 17.25; 35.75)	17.40	11.784	15 (IQR 8.25; 26.00)	<0.001	0.556
ADS-L	19.43	9.532	17 (IQR 12.25; 24.00)	9.77	8.266	8 (IQR 4.00; 13.00)	<0.001	0.574

SD = Standard Deviation; η^2^ = Eta-squared. * Values are expressed as median and interquartile range (IQR) of the 25th and 75th percentile.

**Table 5:** Associations between dichotomized patients’ perceived treatment helpfulness and changes (Δ) in pain, physical and psychosocial functioning.

Helpfulness of Treatment (Yes/No)	Test	Δ FFbH-R	Δ PDI	Δ ADS-L	Δ Pain Average	Δ Pain Worst	Δ Pain Least	Δ Pain Current
Program (in general)	H (1) =	13.534	7.936	3.044	17.454	19.262	9.031	8.201
	*p*-value	<0.001	0.005	0.081	<0.001	<0.001	0.003	0.004
	η^2^	0.046	0.025	0.007	0.060	0.067	0.029	0.026
Physiotherapy (group)	H (1) =	14.348	13.514	3.171	19.949	14.110	6.610	7.701
	*p*-value	<0.001	<0.001	0.075	<0.001	<0.001	0.010	0.006
	η^2^	0.049	0.046	0.008	0.069	0.048	0.02	0.024
Physiotherapy (individual)	F (1, 274) =	3.347	2.779	0.301	2.102	3.395	0.510	1.284
	*p*-value	0.068	0.097	0.584	0.148	0.066	0.476	0.258
	η	0.012	0.010	0.001	0.008	0.012	0.002	0.005
Relaxation therapy	F (1, 274) =	2.138	3.126	3.494	5.949	6.661	7.883	8.073
	*p*-value	0.145	0.078	0.063	0.015	0.010	0.005	0.005
	η^2^	0.008	0.011	0.013	0.021	0.024	0.028	0.029
Aquatic therapy	H (1) =	4.421	2.200	0.318	2.009	4.032	1.974	3.215
	*p*-value	0.036	0.138	0.573	0.156	0.045	0.160	0.073
	η^2^	0.012	0.004	0.002	0.004	0.011	0.004	0.008
Back education	F (1, 274) =	9.865	2.535	1.217	11.824	13.393	5.559	4.785
	*p*-value	0.002	0.113	0.271	<0.001	<0.001	0.019	0.030
	η^2^	0.035	0.009	0.004	0.041	0.047	0.020	0.017
Music therapy	F (1, 274) =	7.958	1.483	0.004	0.323	2.971	1.889	3.117
	*p*-value	0.005	0.224	0.952	0.570	0.086	0.170	0.079
	η^2^	0.028	0.005	0.000	0.001	0.011	0.007	0.011
Psychological pain therapy	F (1, 274) =	0.510	1.593	0.380	1.943	4.048	1.621	1.553
	*p*-value	0.476	0.208	0.538	0.165	0.045	0.204	0.214
	η^2^	0.002	0.006	0.001	0.007	0.015	0.006	0.006
Biofeedback	F (1, 274) =	8.261	6.592	0.115	8.851	8.691	5.079	4.573
	*p*-value	0.004	0.011	0.735	0.003	0.003	0.025	0.033
	η^2^	0.029	0.023	0.000	0.031	0.031	0.018	0.016
Medical training therapy	H (1) =	9.694	6.045	0.029	5.817	10.830	1.249	1.160
	*p*-value	0.002	0.014	0.865	0.016	0.001	0.264	0.281

H = Kruskal–Wallis Test; F = one-way multivariate analysis of variance (MANOVA, with Bonferroni adjustment for multiple comparisons); H-tests instead of F-tests were used if homogeneity of variances was violated. η^2^ = (partial-) Eta-squared; FFbH-R = Hannover Functional Ability Questionnaire; PDI = Pain Disability Index; ADS-L = German Version of the Center for Epidemiologic Studies Depression Scale.

## Supplementary Materials

The following are available online at https://www.mdpi.com/2077-0383/9/1/145/s1, Table S1: Patients’ perceived helpfulness of treatments at discharge; Table S2: Patients’ perceived helpfulness of treatments at discharge and frequencies of dichotomized values; Table S3: Correlations between the patients’ perceived helpfulness and sociodemographic/pain-related characteristics; Table S4: Wilcoxon signed-rank test—differences of dependent variables between study entry and discharge after three weeks; Table S5: Changes (Delta = Δ) between T1 and T0 for pain intensities, physical and psychological functioning; Table S6: Kruskal–Wallis H-Test for changes (delta) in pain, physical and psychosocial function grouped by dichotomized patients’ perceived treatment helpfulness; Table S7: MANOVA-related descriptive statistics for changes (Δ = delta) in pain, physical and psychosocial functioning grouped by dichotomized patients’ perceived treatment helpfulness; Table S8: MANOVA pairwise comparisons of changes (Δ = delta values) in pain, physical and psychosocial functioning by dichotomized patients’ perceived treatment helpfulness.
