# Supplementary material for: Multidisciplinary Pain Management of Chronic Back Pain: Helpful Treatments from the Patients’ Perspective
Source: J Clin Med. 2020 Jan 5;9(1):145. doi: 10.3390/jcm9010145 (PMC7019713; doi:10.3390/jcm9010145)
Supplement: Supplementary file 1 [file jcm-09-00145-s001.zip › jcm-660652suppl/Table S1.docx]

| **Table S1**. Patients' perceived helpfulness of treatments at discharge (n=276). Treatments were considered helpful when rated ≥ 4 (= moderately helpful). | | | | | | |
| --- | --- | --- | --- | --- | --- | --- |
| **Treatment** | **not at all helpful** | **I don't know** | **slightly helpful** | **moderately helpful** | **really helpful** | **extremly helpful** |
| Program (in general) | 8 | 0 | 60 | 83 | 57 | 68 |
| Physiotherapy (group) | 3 | 4 | 28 | 62 | 73 | 106 |
| Physiotherapy (individual) | 3 | 5 | 32 | 42 | 60 | 134 |
| Relaxation therapy | 6 | 4 | 50 | 66 | 58 | 92 |
| Aquatic therapy | 12 | 9 | 40 | 59 | 70 | 86 |
| Back education | 6 | 14 | 49 | 65 | 69 | 73 |
| Medical training therapy | 51 | 13 | 83 | 62 | 42 | 25 |
| Biofeedback | 44 | 22 | 104 | 46 | 31 | 15 |
| Psychological pain therapy | 54 | 17 | 105 | 50 | 34 | 16 |
| Music therapy | 66 | 19 | 96 | 49 | 31 | 15 |
