# Supplementary material for: Multidisciplinary Pain Management of Chronic Back Pain: Helpful Treatments from the Patients’ Perspective
Source: J Clin Med. 2020 Jan 5;9(1):145. doi: 10.3390/jcm9010145 (PMC7019713; doi:10.3390/jcm9010145)
Supplement: Supplementary file 1 [file jcm-09-00145-s001.zip › jcm-660652suppl/Table S2 .docx]

| **Table S2.** Patients' perceived helpfulness of treatments at discharge (N=276) and frequencies of dichotomized values. Treatments were considered helpful when rated ≥ 4 (= moderately helpful). | | | | | | |
| --- | --- | --- | --- | --- | --- | --- |
| **Treatments** | **Response** | **Mean** | **SD** | **SEM** | **Frequency** | **Percent** |
| Program (in general) |  | 4.39 | 1.23 | 0.074 |  |  |
|  | yes |  |  |  | 208 | 75.4 |
|  | no |  |  |  | 68 | 24.6 |
| Physiotherapy (group) |  | 4.87 | 1.14 | 0.069 |  |  |
|  | yes |  |  |  | 241 | 87.3 |
|  | no |  |  |  | 35 | 12.7 |
| Physiotherapy (individual) |  | 5.00 | 1.20 | 0.072 |  |  |
|  | yes |  |  |  | 236 | 85.5 |
|  | no |  |  |  | 40 | 14.5 |
| Relaxation therapy |  | 4.60 | 1.27 | 0.077 |  |  |
|  | yes |  |  |  | 216 | 78.3 |
|  | no |  |  |  | 60 | 21.7 |
| Aquatic therapy |  | 4.54 | 1.37 | 0.083 |  |  |
|  | yes |  |  |  | 215 | 77.9 |
|  | no |  |  |  | 61 | 22.1 |
| Back education |  | 4.43 | 1.30 | 0.079 |  |  |
|  | yes |  |  |  | 207 | 75.0 |
|  | no |  |  |  | 69 | 25.0 |
| Medical training therapy |  | 3.38 | 1.52 | 0.091 |  |  |
|  | yes |  |  |  | 147 | 53.3 |
|  | no |  |  |  | 129 | 46.7 |
| Biofeedback therapy |  | 3.31 | 1.48 | 0.089 |  |  |
|  | yes |  |  |  | 170 | 61.6 |
|  | no |  |  |  | 106 | 38.4 |
| Psychological pain therapy |  | 3.15 | 1.42 | 0.086 |  |  |
|  | yes |  |  |  | 176 | 63.8 |
|  | no |  |  |  | 100 | 36.2 |
| Music therapy |  | 3.02 | 1.47 | 0.088 |  |  |
|  | yes |  |  |  | 181 | 65.6 |
|  | no |  |  |  | 95 | 34.4 |
| SD = standard deviation; SEM = standard error of mean | | | | | | |
