# Supplementary material for: Multidisciplinary Pain Management of Chronic Back Pain: Helpful Treatments from the Patients’ Perspective
Source: J Clin Med. 2020 Jan 5;9(1):145. doi: 10.3390/jcm9010145 (PMC7019713; doi:10.3390/jcm9010145)
Supplement: Supplementary file 1 [file jcm-09-00145-s001.zip › jcm-660652suppl/Table S3.docx]

| **Table S3.** Correlations between the patients' perceived helpfulness and socio-demographic/pain-related characteristics. | | | | | | | | | | | | |
| --- | --- | --- | --- | --- | --- | --- | --- | --- | --- | --- | --- | --- |
|  | **Program (in general)** | | **Psychological pain therapy** | | **Medical training therapy** | | **Music therapy** | | **Aquatic therapy** | | **Back education** | |
|  | r_s_ | p | r_s_ | p | r_s_ | p | r_s_ | p | r_s_ | p | r_s_ | p |
| Education |  |  | -0.153 | 0.011 | 0.133 | 0.028 | -0.132 | 0.029 |  |  |  |  |
| Age |  |  | 0.147 | 0.015 | -0.189 | 0.002 |  |  |  |  |  |  |
| Sports Activity |  |  |  |  | -0.139 | 0.021 |  |  |  |  |  |  |
| BMI |  |  | 0.122 | 0.043 |  |  |  |  | 0.148 | 0.014 |  |  |
| Pain chronicity | -0.158 | 0.009 |  |  |  |  |  |  |  |  | -0.120 | 0.046 |
| r_s_= Spearman's rho; p= significance value (two tailed) | | | | | | | | | | | | |
