# Supplementary material for: Multidisciplinary Pain Management of Chronic Back Pain: Helpful Treatments from the Patients’ Perspective
Source: J Clin Med. 2020 Jan 5;9(1):145. doi: 10.3390/jcm9010145 (PMC7019713; doi:10.3390/jcm9010145)
Supplement: Supplementary file 1 [file jcm-09-00145-s001.zip › jcm-660652suppl/Table S4.docx]

| **Table S4.** Wilcoxon signed-rank test – differences of dependent variables between study entry and discharge after three weeks | | | | | | | |
| --- | --- | --- | --- | --- | --- | --- | --- |
|  |  |  | Perzentile | | |  |  |
|  | Mean | SD | 25. | 50. (Median) | 75. | Z | p-value |
| analgesic intake T1 | 2.27 | 1.136 | 1.00 | 2.00 | 3.00 | -0.020 | 0.984 |
| analgesic intake T2 | 2.27 | 1.234 | 1.00 | 2.00 | 4.00 |  |  |
|  |  |  |  |  |  |  |  |
| average pain last week T1 | 5.24 | 2.079 | 4.00 | 5.00 | 7.00 | -10.164 | < 0.001 |
| average pain last week T2 | 3.80 | 2.021 | 2.00 | 4.00 | 5.00 |  |  |
|  |  |  |  |  |  |  |  |
| worst pain T1 | 6.97 | 2.127 | 6.00 | 8.00 | 9.00 | -8.204 | < 0.001 |
| worst painT2 | 5.80 | 2.350 | 4.00 | 6.00 | 8.00 |  |  |
|  |  |  |  |  |  |  |  |
| least pain T1 | 2.77 | 1.930 | 1.00 | 3.00 | 4.00 | -8.042 | < 0.001 |
| least pain T2 | 1.95 | 1.846 | 1.00 | 1.00 | 3.00 |  |  |
|  |  |  |  |  |  |  |  |
| current pain T1 | 4.41 | 2.416 | 3.00 | 4.00 | 6.00 | -9.134 | < 0.001 |
| current pain T2 | 3.07 | 2.374 | 1.00 | 3.00 | 5.00 |  |  |
|  |  |  |  |  |  |  |  |
| FFbHR T1 | 74.54 | 16.887 | 63.00 | 75.00 | 88.00 | -4.198 | < 0.001 |
| FFbHR T2 | 71.49 | 15.070 | 58.00 | 71.00 | 83.00 |  |  |
|  |  |  |  |  |  |  |  |
| PDI T1 | 26.70 | 12.266 | 17.25 | 25.00 | 35.75 | -12.387 | < 0.001 |
| PDI T2 | 17.40 | 11.784 | 8.25 | 15.00 | 26.00 |  |  |
|  |  |  |  |  |  |  |  |
| ADS T1 | 19.43 | 9.532 | 12.25 | 17.00 | 24.00 | -12.592 | < 0.001 |
| ADS T2 | 9.77 | 8.266 | 4.00 | 8.00 | 13.00 |  |  |
| SD = standard deviation. FFbH-R=Hannover Functional Ability Questionnaire; PDI=Pain Disability Index; ADS-L=German Version of the Center for Epidemiologic Studies Depression Scale | | | | | | | |
