# Supplementary material for: Multidisciplinary Pain Management of Chronic Back Pain: Helpful Treatments from the Patients’ Perspective
Source: J Clin Med. 2020 Jan 5;9(1):145. doi: 10.3390/jcm9010145 (PMC7019713; doi:10.3390/jcm9010145)
Supplement: Supplementary file 1 [file jcm-09-00145-s001.zip › jcm-660652suppl/Table S5.docx]

| **Table S5.** Changes (Delta = Δ) between T1 and T0 for pain intensities. physical and psychological functioning (N=276). | | | | | | | |
| --- | --- | --- | --- | --- | --- | --- | --- |
|  |  |  |  |  |  | **Percentiles** |  |
| Delta = Δ | **Mean** | **SD** | **Min** | **Max** | **25th** | **50th (Median)** | **75th** |
| Δ FFbH-R* | -3.05 | 19.729 | -65 | 79 | -16.00 | -4.00 | 4.00 |
| Δ PDI | 9.30 | 9.763 | -20 | 53 | 2.00 | 8.00 | 15.00 |
| Δ ADS-L | 9.66 | 9.141 | -19 | 42 | 4.00 | 10.00 | 15.00 |
| Δ Pain average | 1.43 | 1.937 | -6 | 7 | 0.00 | 1.00 | 3.00 |
| Δ Pain worst | 1.17 | 2.126 | -5 | 9 | 0.00 | 1.00 | 2.00 |
| Δ Pain least | 0.82 | 1.650 | -9 | 7 | 0.00 | 1.00 | 2.00 |
| Δ Pain current | 1.34 | 2.207 | -7 | 9 | 0.00 | 1.00 | 3.00 |
| * Result=T1-T0. other Results=T0-T1 for expressing improvements as positive value; FFbH-R=Hannover Functional Ability Questionnaire; PDI=Pain Disability Index; ADS-L=German Version of the Center for Epidemiologic Studies Depression Scale. | | | | | | | |
