# Supplementary material for: Multidisciplinary Pain Management of Chronic Back Pain: Helpful Treatments from the Patients’ Perspective
Source: J Clin Med. 2020 Jan 5;9(1):145. doi: 10.3390/jcm9010145 (PMC7019713; doi:10.3390/jcm9010145)
Supplement: Supplementary file 1 [file jcm-09-00145-s001.zip › jcm-660652suppl/Table S6.docx]

| **Table S6.** Kruskal-Wallis H-Test for changes (delta) in pain, physical and psychosocial function grouped by dichotomized patients' perceived treatment helpfulness (N = 276) | | | | | | | | | | | | | | | | | | | | | | | | | | | | | | | | | | | | | | | | | | | | | | | | | | | | | | | |  |
| --- | --- | --- | --- | --- | --- | --- | --- | --- | --- | --- | --- | --- | --- | --- | --- | --- | --- | --- | --- | --- | --- | --- | --- | --- | --- | --- | --- | --- | --- | --- | --- | --- | --- | --- | --- | --- | --- | --- | --- | --- | --- | --- | --- | --- | --- | --- | --- | --- | --- | --- | --- | --- | --- | --- | --- | --- |
| **program (in general) helpful (yes/no)** | | | | | | | | | | | | | | | | | | | | | | | | | | | | | | | | | | | | | | | | | | | | | | | | | | | | | | | |  |
|  | | | | **FFbH-R** | | | | | | | | **PDI** | | | | | | | | **ADS-L** | | | | | | | | **Pain average** | | | | | **Pain worst** | | | | | | | | **Pain least** | | | | | | | | **Pain current** | | | | | | |  |
|  | | | | **No** | | | | **Yes** | | | | **No** | | | | **Yes** | | | | **No** | | | | **Yes** | | | | **No** | | **Yes** | | | **No** | | | | **Yes** | | | | **No** | | | **Yes** | | | | | **No** | | | **Yes** | | | |  |
| Frequency | | | | 68 | | | | 208 | | | | 68 | | | | 208 | | | | 68 | | | | 208 | | | | 68 | | 208 | | | 68 | | | | 208 | | | | 68 | | | 208 | | | | | 68 | | | 208 | | | |  |
| Mean Rank | | | | 169.26 | | | | 128.44 | | | | 114.85 | | | | 146.23 | | | | 123.85 | | | | 143.29 | | | | 103.97 | | 149.79 | | | 102.07 | | | | 150.41 | | | | 113.95 | | | 146.53 | | | | | 114.74 | | | 146.27 | | | |  |
| H (df =1) | | | | 13.534 | | | | | | | | 7.936 | | | | | | | | 3.044 | | | | | | | | 17.454 | | | | | 19.262 | | | | | | | | 9.031 | | | | | | | | 8.201 | | | | | | |  |
| **physiotherapy (group) helpful (yes/no)** | | | | | | | | | | | | | | | | | | | | | | | | | | | | | | | | | | | | | | | | | | | | | | | | | | | | | | | |  |
|  | **FFbH-R** | | | | | | | | **PDI** | | | | | | | | **ADS-L** | | | | | | | | **Pain average** | | | | | | | **Pain worst** | | | | | | | | **Pain least** | | | | | | | | **Pain current** | | | | | | | |  |
|  | **No** | | | | **Yes** | | | | **No** | | | | **Yes** | | | | **No** | | | | **Yes** | | | | **No** | | | | **Yes** | | | **No** | | | | **Yes** | | | | **No** | | | | | **Yes** | | | **No** | | | | | **Yes** | | |  |
| Frequency | 35 | | | | 241 | | | | 35 | | | | 241 | | | | 35 | | | | 241 | | | | 35 | | | | 241 | | | 35 | | | | 241 | | | | 35 | | | | | 241 | | | 35 | | | | | 241 | | |  |
| Mean Rank | 186.01 | | | | 131.60 | | | | 92.19 | | | | 145.23 | | | | 116.07 | | | | 141.76 | | | | 83.11 | | | | 146.54 | | | 91.71 | | | | 145.29 | | | | 106.99 | | | | | 143.08 | | | 103.96 | | | | | 143.52 | | |  |
| H (df =1) | 14.348 | | | | | | | | 13.514 | | | | | | | | 3.171 | | | | | | | | 19.949 | | | | | | | 14.110 | | | | | | | | 6.610 | | | | | | | | 7.701 | | | | | | | |  |
| **medical training therapy helpful (yes/no)** | | | | | | | | | | | | | | | | | | | | | | | | | | | | | | | | | | | | | | | | | | | | | | | | | | | | | | | | |
|  | | **FFbH-R** | | | | | | | | **PDI** | | | | | | | | | **ADS-L** | | | | | | | **Pain average** | | | | | | | | **Pain worst** | | | | | | | | | **Pain least** | | | | | | | **Pain current** | | | | | | |
|  | | **No** | | | | **Yes** | | | | **No** | | | | **Yes** | | | | | **No** | | | **Yes** | | | | **No** | | | | **Yes** | | | | **No** | | | | **Yes** | | | | | **No** | | | **Yes** | | | | **No** | | | | **Yes** | | |
| Frequency | | 147 | | | | 129 | | | | 147 | | | | 129 | | | | | 147 | | | 129 | | | | 147 | | | | 129 | | | | 147 | | | | 129 | | | | | 147 | | | 129 | | | | 147 | | | | 129 | | |
| Mean Rank | | 152.44 | | | | 122.61 | | | | 127.44 | | | | 151.10 | | | | | 137.73 | | | 139.37 | | | | 127.82 | | | | 150.67 | | | | 123.87 | | | | 155.17 | | | | | 133.61 | | | 144.07 | | | | 133.71 | | | | 143.95 | | |
| H (df =1) | | 9.694 | | | | | | | | 6.045 | | | | | | | | | 0.029 | | | | | | | 5.817 | | | | | | | | 10.830 | | | | | | | | | 1.249 | | | | | | | 1.160 | | | | | | |
| **aquatic therapy helpful (yes/no)** | | | | | | | | | | | | | | | | | | | | | | | | | | | | | | | | | | | | | | | | | | | | | | | | | | | | | | | | |
|  | | | **FFbH-R** | | | | | | | | **PDI** | | | | | | | **ADS-L** | | | | | | | | | **Pain average** | | | | | | | | **Pain worst** | | | | | | | **Pain least** | | | | | | | | | **Pain current** | | | | | |
|  | | | **No** | | | | **Yes** | | | | **No** | | | | **Yes** | | | **No** | | | | | **Yes** | | | | **No** | | | | **Yes** | | | | **No** | | | | **Yes** | | | **No** | | | | | **Yes** | | | | **No** | | | | **Yes** | |
| Frequency | | | 61 | | | | 215 | | | | 61 | | | | 215 | | | 61 | | | | | 215 | | | | 61 | | | | 215 | | | | 61 | | | | 215 | | | 61 | | | | | 215 | | | | 61 | | | | 215 | |
| Mean Rank | | | 157.37 | | | | 133.15 | | | | 125.13 | | | | 142.29 | | | 143.58 | | | | | 137.06 | | | | 125.93 | | | | 142.07 | | | | 120.61 | | | | 143.58 | | | 126.18 | | | | | 142.00 | | | | 122.53 | | | | 143.03 | |
| H (df =1) | | | 4.421 | | | | | | | | 2.200 | | | | | | | 0.318 | | | | | | | | | 2.009 | | | | | | | | 4.032 | | | | | | | 1.974 | | | | | | | | | 3.215 | | | | | |
| H = Kruskal Wallis-Test; df = degrees of freedom | | | | | | | | | | | | | | | | | | | | | | | | | | | | | | | | | | | | | | | | | | | | | | | | | | | | | | | | |
|  | | | | | | | | | | | | | | | | | | | | | | | | | | | | | | | | | | | | | | | | | | | | | | | | | | | | | | | | |
