# Supplementary material for: Multidisciplinary Pain Management of Chronic Back Pain: Helpful Treatments from the Patients’ Perspective
Source: J Clin Med. 2020 Jan 5;9(1):145. doi: 10.3390/jcm9010145 (PMC7019713; doi:10.3390/jcm9010145)
Supplement: Supplementary file 1 [file jcm-09-00145-s001.zip › jcm-660652suppl/Table S7 .docx]

**Table S7**. MANOVA related descriptive statistics for changes (Δ = delta) in pain, physical and psychosocial functioning grouped by dichotomized patients' perceived treatment helpfulness

|  | **Physiotherapy individual**  **helpful (Yes/No)?** | **N** | **Mean** | **Std. Deviation** | **95% - CI lower bound upper bound** | |
| --- | --- | --- | --- | --- | --- | --- |
| Δ FFbH-R | No | 40 | 2.20 | 22.393 | -3.915 | 8.315 |
|  | Yes | 236 | -3.94 | 19.151 | -6.462 | -1.427 |
|  | Total | 276 | -3.05 | 19.729 |  |  |
| Δ PDI | No | 40 | 6.93 | 8.971 | 3.896 | 9.954 |
|  | Yes | 236 | 9.70 | 9.852 | 8.452 | 10.946 |
|  | Total | 276 | 9.30 | 9.763 |  |  |
| Δ ADS-L | No | 40 | 8.93 | 9.799 | 6.076 | 11.774 |
|  | Yes | 236 | 9.78 | 9.041 | 8.611 | 10.957 |
|  | Total | 276 | 9.66 | 9.141 |  |  |
| Δ Pain average | No | 40 | 1.03 | 2.006 | 0.423 | 1.627 |
|  | Yes | 236 | 1.50 | 1.921 | 1.257 | 1.752 |
|  | Total | 276 | 1.43 | 1.937 |  |  |
| Δ Pain worst | No | 40 | 0.60 | 1.905 | -0.059 | 1.259 |
|  | Yes | 236 | 1.27 | 2.150 | 0.996 | 1.538 |
|  | Total | 276 | 1.17 | 2.126 |  |  |
| Δ Pain least | No | 40 | 0.65 | 1.388 | 0.136 | 1.164 |
|  | Yes | 236 | 0.85 | 1.691 | 0.640 | 1.063 |
|  | Total | 276 | 0.82 | 1.650 |  |  |
| Δ Pain current | No | 40 | 0.98 | 1.981 | 0.288 | 1.662 |
|  | Yes | 236 | 1.40 | 2.242 | 1.120 | 1.685 |
|  | Total | 276 | 1.34 | 2.207 |  |  |
|  | **Relaxation therapy helpful (Yes/No)?** | **N** | **Mean** | **Std. Deviation** | **95% - CI lower bound upper bound** | |
| Δ FFbH-R | No | 60 | 0.23 | 20.711 | -4.770 | 5.237 |
|  | Yes | 216 | -3.97 | 19.398 | -6.605 | -1.330 |
|  | Total | 276 | -3.05 | 19.729 |  |  |
| Δ PDI | No | 60 | 7.33 | 10.741 | 4.862 | 9.805 |
|  | Yes | 216 | 9.84 | 9.427 | 8.540 | 11.145 |
|  | Total | 276 | 9.30 | 9.763 |  |  |
| Δ ADS-L | No | 60 | 7.72 | 9.394 | 5.404 | 10.029 |
|  | Yes | 216 | 10.20 | 9.017 | 8.980 | 11.418 |
|  | Total | 276 | 9.66 | 9.141 |  |  |
| Δ Pain average | No | 60 | 0.90 | 1.928 | 0.412 | 1.388 |
|  | Yes | 216 | 1.58 | 1.917 | 1.326 | 1.840 |
|  | Total | 276 | 1.43 | 1.937 |  |  |
| Δ Pain worst | No | 60 | 0.55 | 2.086 | 0.015 | 1.085 |
|  | Yes | 216 | 1.34 | 2.109 | 1.061 | 1.624 |
|  | Total | 276 | 1.17 | 2.126 |  |  |
| Δ Pain least | No | 60 | 0.30 | 1.453 | -0.114 | 0.714 |
|  | Yes | 216 | 0.97 | 1.674 | 0.749 | 1.186 |
|  | Total | 276 | 0.82 | 1.650 |  |  |
| Δ Pain current | No | 60 | 0.63 | 1.746 | 0.079 | 1.187 |
|  | Yes | 216 | 1.54 | 2.284 | 1.245 | 1.829 |
|  | Total | 276 | 1.34 | 2.207 |  |  |
|  | **Psychological pain therapy helpful (Yes/No)?** | **N** | **Mean** | **Std. Deviation** | **95% - CI lower bound upper bound** | |
| Δ FFbH-R | No | 176 | -2.41 | 20.470 | -5.345 | 0.515 |
|  | Yes | 100 | -4.18 | 18.397 | -8.067 | -0.293 |
|  | Total | 276 | -3.05 | 19.729 |  |  |
| Δ PDI | No | 176 | 8.74 | 9.207 | 7.291 | 10.186 |
|  | Yes | 100 | 10.28 | 10.648 | 8.360 | 12.200 |
|  | Total | 276 | 9.30 | 9.763 |  |  |
| Δ ADS-L | No | 176 | 9.40 | 8.166 | 8.045 | 10.761 |
|  | Yes | 100 | 10.11 | 10.672 | 8.308 | 11.912 |
|  | Total | 276 | 9.66 | 9.141 |  |  |
| Δ Pain average | No | 176 | 1.31 | 1.885 | 1.026 | 1.599 |
|  | Yes | 100 | 1.65 | 2.017 | 1.269 | 2.031 |
|  | Total | 276 | 1.43 | 1.937 |  |  |
| Δ Pain worst | No | 176 | 0.98 | 2.062 | 0.664 | 1.291 |
|  | Yes | 100 | 1.51 | 2.204 | 1.094 | 1.926 |
|  | Total | 276 | 1.17 | 2.126 |  |  |
| Δ Pain least | No | 176 | 0.73 | 1.591 | 0.483 | 0.972 |
|  | Yes | 100 | 0.99 | 1.744 | 0.666 | 1.314 |
|  | Total | 276 | 0.82 | 1.650 |  |  |
| Δ Pain current | No | 176 | 1.22 | 2.143 | 0.889 | 1.543 |
|  | Yes | 100 | 1.56 | 2.311 | 1.126 | 1.994 |
|  | Total | 276 | 1.34 | 2.207 |  |  |
|  | **Biofeedback helpful (Yes/No)?** | **N** | **Mean** | **Std. Deviation** | **95% - CI lower bound upper bound** | |
| Δ FFbH-R | No | 170 | -0.39 | 20.095 | -3.334 | 2.546 |
|  | Yes | 106 | -7.32 | 18.429 | -11.044 | -3.597 |
|  | Total | 276 | -3.05 | 19.729 |  |  |
| Δ PDI | No | 170 | 8.12 | 9.083 | 6.658 | 9.577 |
|  | Yes | 106 | 11.19 | 10.534 | 9.341 | 13.037 |
|  | Total | 276 | 9.30 | 9.763 |  |  |
| Δ ADS-L | No | 170 | 9.51 | 8.786 | 8.129 | 10.894 |
|  | Yes | 106 | 9.90 | 9.721 | 8.146 | 11.647 |
|  | Total | 276 | 9.66 | 9.141 |  |  |
| Δ Pain average | No | 170 | 1.16 | 1.890 | 0.876 | 1.453 |
|  | Yes | 106 | 1.87 | 1.942 | 1.503 | 2.233 |
|  | Total | 276 | 1.43 | 1.937 |  |  |
| Δ Pain worst | No | 170 | 0.88 | 2.053 | 0.560 | 1.193 |
|  | Yes | 106 | 1.64 | 2.165 | 1.241 | 2.042 |
|  | Total | 276 | 1.17 | 2.126 |  |  |
| Δ Pain least | No | 170 | 0.65 | 1.759 | 0.400 | 0.894 |
|  | Yes | 106 | 1.10 | 1.420 | 0.791 | 1.417 |
|  | Total | 276 | 0.82 | 1.650 |  |  |
| Δ Pain current | No | 170 | 1.12 | 2.245 | 0.786 | 1.449 |
|  | Yes | 106 | 1.70 | 2.107 | 1.279 | 2.117 |
|  | Total | 276 | 1.34 | 2.207 |  |  |
|  | **Music therapy helpful (Yes/No)?** | **N** | **Mean** | **Std. Deviation** | **95% - CI lower bound upper bound** | |
| Δ FFbH-R | No | 181 | -0.66 | 20.999 | -3.508 | 2.194 |
|  | Yes | 95 | -7.62 | 16.191 | -11.556 | -3.686 |
|  | Total | 276 | -3.05 | 19.729 |  |  |
| Δ PDI | No | 181 | 8.78 | 9.452 | 7.352 | 10.206 |
|  | Yes | 95 | 10.28 | 10.309 | 8.314 | 12.254 |
|  | Total | 276 | 9.30 | 9.763 |  |  |
| Δ ADS-L | No | 181 | 9.64 | 9.166 | 8.295 | 10.975 |
|  | Yes | 95 | 9.71 | 9.141 | 7.856 | 11.555 |
|  | Total | 276 | 9.66 | 9.141 |  |  |
| Δ Pain average | No | 181 | 1.39 | 1.869 | 1.103 | 1.671 |
|  | Yes | 95 | 1.53 | 2.067 | 1.135 | 1.918 |
|  | Total | 276 | 1.43 | 1.937 |  |  |
| Δ Pain worst | No | 181 | 1.01 | 2.025 | 0.701 | 1.321 |
|  | Yes | 95 | 1.47 | 2.287 | 1.046 | 1.902 |
|  | Total | 276 | 1.17 | 2.126 |  |  |
| Δ Pain least | No | 181 | 0.72 | 1.581 | 0.483 | 0.965 |
|  | Yes | 95 | 1.01 | 1.765 | 0.678 | 1.343 |
|  | Total | 276 | 0.82 | 1.650 |  |  |
| Δ Pain current | No | 181 | 1.17 | 2.068 | 0.849 | 1.493 |
|  | Yes | 95 | 1.66 | 2.430 | 1.219 | 2.107 |
|  | Total | 276 | 1.34 | 2.207 |  |  |
|  | **Back education helpful (Yes/No)?** | **N** | **Mean** | **Std. Deviation** | **95% - CI lower bound upper bound** | |
| Δ FFbH-R | No | 69 | 3.30 | 23.018 | -1.298 | 7.906 |
|  | Yes | 207 | -5.17 | 18.073 | -7.831 | -2.517 |
|  | Total | 276 | -3.05 | 19.729 |  |  |
| Δ PDI | No | 69 | 7.68 | 9.486 | 5.374 | 9.989 |
|  | Yes | 207 | 9.84 | 9.817 | 8.504 | 11.168 |
|  | Total | 276 | 9.30 | 9.763 |  |  |
| Δ ADS-L | No | 69 | 8.61 | 8.999 | 6.443 | 10.774 |
|  | Yes | 207 | 10.01 | 9.183 | 8.759 | 11.260 |
|  | Total | 276 | 9.66 | 9.141 |  |  |
| Δ Pain average | No | 69 | 0.75 | 1.794 | 0.303 | 1.204 |
|  | Yes | 207 | 1.66 | 1.934 | 1.402 | 1.922 |
|  | Total | 276 | 1.43 | 1.937 |  |  |
| Δ Pain worst | No | 69 | 0.38 | 1.964 | -0.116 | 0.870 |
|  | Yes | 207 | 1.43 | 2.117 | 1.150 | 1.719 |
|  | Total | 276 | 1.17 | 2.126 |  |  |
| Δ Pain least | No | 69 | 0.42 | 1.489 | 0.033 | 0.808 |
|  | Yes | 207 | 0.96 | 1.682 | 0.733 | 1.180 |
|  | Total | 276 | 0.82 | 1.650 |  |  |
| Δ Pain current | No | 69 | 0.84 | 2.266 | 0.321 | 1.360 |
|  | Yes | 207 | 1.51 | 2.167 | 1.207 | 1.807 |
|  | Total | 276 | 1.34 | 2.207 |  |  |

FFbH-R=Hannover Functional Ability Questionnaire; PDI=Pain Disability Index; ADS-L=German Version of the Center for Epidemiologic Studies Depression Scale
