# Supplementary material for: Multidisciplinary Pain Management of Chronic Back Pain: Helpful Treatments from the Patients’ Perspective
Source: J Clin Med. 2020 Jan 5;9(1):145. doi: 10.3390/jcm9010145 (PMC7019713; doi:10.3390/jcm9010145)
Supplement: Supplementary file 1 [file jcm-09-00145-s001.zip › jcm-660652suppl/Table S8.docx]

| **Table S8.** MANOVA pairwise comparisons of changes (Δ = delta values) in pain, physical and psychosocial functioning by dichotomized patients' perceived treatment helpfulness | | | | | | | |
| --- | --- | --- | --- | --- | --- | --- | --- |
| **Program in general helpful** | | | | | | | |
|  | T2 helpful (yes/no) | T2 helpful (yes/no) | Mean Difference (I-J) | Std. Error | Sig | 95% Confidence Interval for Difference  Lower Bound Upper Bound | |
| Δ FFbHR | No | Yes | 11.663^*^ | 2.670 | .000 | 6.408 | 16.919 |
| Δ PDI | No | Yes | -3.868^*^ | 1.346 | .004 | -6.518 | -1.218 |
| Δ ADS | No | Yes | -2.026 | 1.273 | .113 | -4.533 | 0.481 |
| Δ Pain average | No | Yes | -0.909^*^ | 0.265 | .001 | -1.431 | -0.386 |
| Δ Pain worst | No | Yes | -1.241^*^ | 0.288 | .000 | -1.808 | -0.674 |
| Δ Pain least | No | Yes | -0.545^*^ | 0.228 | .018 | -0.995 | -0.095 |
| Δ Pain current | No | Yes | -0.862^*^ | 0.305 | .005 | -1.461 | -0.262 |
| **Physiotherapy individual helpful** | | | | | | | |
|  | T2 helpful (yes/no) | T2 helpful (yes/no) | Mean Difference (I-J) | Std. Error | Sig | 95% Confidence Interval for Difference  Lower Bound Upper Bound | |
| Δ FFbHR | No | Yes | 6.145 | 3.359 | 0.068 | -0.468 | 12.758 |
| Δ PDI | No | Yes | -2.774 | 1.664 | 0.097 | -6.050 | 0.502 |
| Δ ADS | No | Yes | -0.859 | 1.565 | 0.584 | -3.940 | 2.222 |
| Δ Pain average | No | Yes | -0.479 | 0.331 | 0.148 | -1.130 | 0.172 |
| Δ Pain worst | No | Yes | -0.667 | 0.362 | 0.066 | -1.380 | 0.046 |
| Δ Pain least | No | Yes | -0.202 | 0.282 | 0.476 | -0.757 | 0.354 |
| Δ Pain current | No | Yes | -0.428 | 0.377 | 0.258 | -1.170 | 0.315 |
| **Physiotherapy group helpful** | | | | | | | |
|  | T2 helpful (yes/no) | T2 helpful (yes/no) | Mean Difference (I-J) | Std. Error | Sig | 95% Confidence Interval for Difference  Lower Bound Upper Bound | |
| Δ FFbHR | No | Yes | 16.423 | 3.435 | 0.000 | 9.661 | 23.185 |
| Δ PDI | No | Yes | -6.721 | 1.722 | 0.000 | -10.111 | -3.331 |
| Δ ADS | No | Yes | -2.915 | 1.647 | 0.078 | -6.157 | 0.328 |
| Δ Pain average | No | Yes | -1.414 | 0.340 | 0.001 | -2.084 | -0.744 |
| Δ Pain worst | No | Yes | -1.406 | 0.376 | 0.000 | -2.146 | -0.666 |
| Δ Pain least | No | Yes | -0.713 | 0.296 | 0.017 | -1.295 | -0.131 |
| Δ Pain current | No | Yes | -1.077 | 0.395 | 0.007 | -1.854 | -0.300 |
| **Relaxation therapy helpful** | | | | | | | |
|  | T2 helpful (yes/no) | T2 helpful (yes/no) | Mean Difference (I-J) | Std. Error | Sig | 95% Confidence Interval for Difference  Lower Bound Upper Bound | |
| Δ FFbHR | No | Yes | 4.201 | 2.873 | 0.145 | -1.455 | 9.857 |
| Δ PDI | No | Yes | -2.509 | 1.419 | 0.078 | -5.303 | 0.285 |
| Δ ADS | No | Yes | -2.482 | 1.328 | 0.063 | -5.097 | 0.132 |
| Δ Pain average | No | Yes | -0.683 | 0.280 | 0.015 | -1.235 | -0.132 |
| Δ Pain worst | No | Yes | -0.793 | 0.307 | 0.010 | -1.397 | -0.188 |
| Δ Pain least | No | Yes | -0.668 | 0.238 | 0.005 | -1.136 | -0.200 |
| Δ Pain current | No | Yes | -0.904 | 0.318 | 0.005 | -1.530 | -0.278 |
| **Medical training helpful** | | | | | | | |
|  | T2 helpful (yes/no) | T2 helpful (yes/no) | Mean Difference (I-J) | Std. Error | Sig | 95% Confidence Interval for Difference  Lower Bound Upper Bound | |
| Δ FFbHR | No | Yes | 7.641 | 2.339 | 0.001 | 3.036 | 12.246 |
| Δ PDI | No | Yes | -3.270 | 1.163 | 0.005 | -5.560 | -0.980 |
| Δ ADS | No | Yes | -0.363 | 1.105 | 0.743 | -2.537 | 1.812 |
| Δ Pain average | No | Yes | -0.523 | 0.232 | 0.025 | -0.979 | -0.066 |
| Δ Pain worst | No | Yes | -0.786 | 0.253 | 0.002 | -1.284 | -0.289 |
| Δ Pain least | No | Yes | -0.086 | 0.199 | 0.667 | -0.478 | 0.306 |
| Δ Pain current | No | Yes | -0.205 | 0.267 | 0.443 | -0.729 | 0.320 |
| **Aquatic therapy helpful** | | | | | | | |
|  | T2 helpful (yes/no) | T2 helpful (yes/no) | Mean Difference (I-J) | Std. Error | Sig | 95% Confidence Interval for Difference  Lower Bound Upper Bound | |
| Δ FFbHR | No | Yes | 7.583 | 2.830 | 0.008 | 2.011 | 13.155 |
| Δ PDI | No | Yes | -2.275 | 1.412 | 0.108 | -5.056 | 0.505 |
| Δ ADS | No | Yes | 1.132 | 1.327 | 0.394 | -1.480 | 3.744 |
| Δ Pain average | No | Yes | -0.369 | 0.281 | 0.190 | -0.921 | 0.184 |
| Δ Pain worst | No | Yes | -0.471 | 0.308 | 0.127 | -1.077 | 0.135 |
| Δ Pain least | No | Yes | -0.298 | 0.239 | 0.213 | -0.769 | 0.172 |
| Δ Pain current | No | Yes | -0.269 | 0.320 | 0.402 | -0.900 | 0.362 |
| **Psychological pain-therapy helpful** | | | | | | | |
|  | T2 helpful (yes/no) | T2 helpful (yes/no) | Mean Difference (I-J) | Std. Error | Sig | 95% Confidence Interval for Difference  Lower Bound Upper Bound | |
| Δ FFbHR | No | Yes | 1.765 | 2.473 | 0.476 | -3.103 | 6.633 |
| Δ PDI | No | Yes | -1.541 | 1.221 | 0.208 | -3.946 | 0.863 |
| Δ ADS | No | Yes | -0.707 | 1.146 | 0.538 | -2.963 | 1.549 |
| Δ Pain average | No | Yes | -0.338 | 0.242 | 0.165 | -0.814 | 0.139 |
| Δ Pain worst | No | Yes | -0.533 | 0.265 | 0.045 | -1.054 | -0.011 |
| Δ Pain least | No | Yes | -0.263 | 0.206 | 0.204 | -0.669 | 0.143 |
| Δ Pain current | No | Yes | -0.344 | 0.276 | 0.214 | -0.888 | 0.200 |
| **Biofeedback helpful** | | | | | | | |
|  | T2 helpful (yes/no) | T2 helpful (yes/no) | Mean Difference (I-J) | Std. Error | Sig | 95% Confidence Interval for Difference  Lower Bound Upper Bound | |
| Δ FFbHR | No | Yes | 6.927 | 2.410 | 0.004 | 2.182 | 11.671 |
| Δ PDI | No | Yes | -3.071 | 1.196 | 0.011 | -5.426 | -0.716 |
| Δ ADS | No | Yes | -0.384 | 1.133 | 0.735 | -2.615 | 1.846 |
| Δ Pain average | No | Yes | -0.703 | 0.236 | 0.003 | -1.169 | -0.238 |
| Δ Pain worst | No | Yes | -0.765 | 0.260 | 0.003 | -1.276 | -0.254 |
| Δ Pain least | No | Yes | -0.457 | 0.203 | 0.025 | -0.856 | -0.058 |
| Δ Pain current | No | Yes | -0.580 | 0.271 | 0.033 | -1.115 | -0.046 |
| **Music therapy helpful** | | | | | | | |
|  | T2 helpful (yes/no) | T2 helpful (yes/no) | Mean Difference (I-J) | Std. Error | Sig | 95% Confidence Interval for Difference  Lower Bound Upper Bound | |
| Δ FFbHR | No | Yes | 6.964 | 2.468 | 0.005 | 2.104 | 11.823 |
| Δ PDI | No | Yes | -1.505 | 1.236 | 0.224 | -3.938 | 0.928 |
| Δ ADS | No | Yes | -0.070 | 1.160 | 0.952 | -2.354 | 2.214 |
| Δ Pain average | No | Yes | -0.140 | 0.246 | 0.570 | -0.623 | 0.344 |
| Δ Pain worst | No | Yes | -0.463 | 0.268 | 0.086 | -0.991 | 0.066 |
| Δ Pain least | No | Yes | -0.287 | 0.209 | 0.170 | -0.698 | 0.124 |
| Δ Pain current | No | Yes | -0.492 | 0.279 | 0.079 | -1.040 | 0.057 |
| **Back education helpful** | | | | | | | |
|  | T2 helpful (yes/no) | T2 helpful (yes/no) | Mean Difference (I-J) | Std. Error | Sig | 95% Confidence Interval for Difference  Lower Bound Upper Bound | |
| Δ FFbHR | No | Yes | 8.478 | 2.699 | 0.002 | 3.164 | 13.792 |
| Δ PDI | No | Yes | -2.155 | 1.353 | 0.113 | -4.819 | 0.510 |
| Δ ADS | No | Yes | -1.401 | 1.270 | 0.271 | -3.902 | 1.100 |
| Δ Pain average | No | Yes | -0.908 | 0.264 | 0.001 | -1.428 | -0.388 |
| Δ Pain worst | No | Yes | -1.058 | 0.289 | 0.000 | -1.627 | -0.489 |
| Δ Pain least | No | Yes | -0.536 | 0.227 | 0.019 | -0.984 | -0.089 |
| Δ Pain current | No | Yes | -0.667 | 0.305 | 0.030 | -1.267 | -0.067 |
| FFbH-R=Hannover Functional Ability Questionnaire; PDI=Pain Disability Index; ADS-L=German Version of the Center for Epidemiologic Studies Depression Scale; Adjustment for multiple comparisons: Bonferroni. | | | | | | | |
